# Two new species of the genus *Melixanthus* Suffrian (Coleoptera, Chrysomelidae, Cryptocephalinae) from China

**DOI:** 10.3897/zookeys.1060.70203

**Published:** 2021-09-17

**Authors:** Wen-Yuan Duan, Feng-Yan Wang, Hong-Zhang Zhou

**Affiliations:** 1 Key Laboratory of Zoological Systematics and Evolution, Institute of Zoology, Chinese Academy of Sciences, 1 Beichen West Rd., Chaoyang District, Beijing 100101, China Institute of Zoology, Chinese Academy of Sciences Beijing China; 2 University of the Chinese Academy of Sciences, 19A Yuquan Rd., Shijingshan District, Beijing 100049, China University of the Chinese Academy of Sciences Beijing China

**Keywords:** Cryptocephalini, distributional records, leaf beetles, new species

## Abstract

Two new species of the genus *Melixanthus* Suffrian, 1854 are described from China: *M.menglaensis* Duan, Wang & Zhou, **sp. nov.** from Yunnan (also in Vietnam, Tonkin) and *M.similibimaculicollis* Duan, Wang & Zhou, **sp. nov.** from Yunnan. Another species, *M.rufiventris* Pic, 1926, is reported for the first time in China. High-quality color images and line drawings of adult habitus, aedeagus, and other important structures are provided for all three species. The types of the new species are deposited in the collection of Institute of Zoology, Chinese Academy of Sciences (IZ-CAS).

## Introduction

The leaf beetle genus *Melixanthus* Suffrian, 1854 (Chrysomelidae, Cryptocephalini) is mainly distributed in the Oriental region and includes approximately 60 species until now; of these, 11 species are known to occur in China (Gressitt and Kimoto 1961; Tan et al. 1980; Tan 1988; Tan and Pu 1992; Lopatin 2005; Schöller et al. 2010). This genus was erected very early on as valid genus-level taxon and can be diagnosed by the following characteristics: antennae rather short, usually reaching humeral region of elytra; apical six segments broadened and flatted, about 1.2–2.2 times as long as wide; claws of all legs usually toothed or thickened basally. Not all *Melixanthus* species readily show the character of claws with or without teeth, and at least a few of species are not easily included in or excluded from this genus. This has challenged taxonomists studying this group.

The Chinese fauna of the genus was studied by including in a comprehensive study on the family Chrysomelidae (e.g., Chûjô 1954; Gressitt and Kimoto 1961; Tan et al. 1980; Schöller et al. 2010). Kimoto and Gressitt (1981) studied the chrysomelid fauna of Thailand, Cambodia, Laos, and Vietnam (countries near China), and thus their publication is important in identifying the Chinese species. The most recent and comprehensive study of *Melixanthus*, having excellent species revisions and key to species, was published by Medvedev (2012).

Our present study reports new findings, including the description of two new species of *Melixanthus* from China.

## Materials and methods

Dried specimens were relaxed in hot distilled water at 80 ℃ for about 2 h to soften the body and ease dissection. The abdomen was separated with insect pins from the rest of the body, soaked in 10% KOH solution and then in a hot water bath for 15 min to advance the process. After this, specimens were transferred in distilled water to rinse the residual KOH solution off and stop the bleaching process. Afterwards, the aedeagus, spermatheca and rectal sclerites were prepared. The dissected parts were placed into glycerin for observation and measurement with an apochromatic stereomicroscope Zeiss SteREO V12. Color photographs of the adults and genitalia were captured with an Axio Zoom V16 fluorescence stereo zoom microscope and photomontage was performed in Zen 2012 (blue edition) imaging software. Adobe Photoshop CS6 was used in digital post-processing of the color images, and Adobe Illustrator 2020 was used to make the line drawings.

Materials used in this study are from the collection: **IZ-CAS** (Institute of Zoology, Chinese Academy of Sciences, Beijing, China).

Measurements are average values calculated from the values of at least five specimens, or all available specimens in case less than five specimens were available. The following abbreviations are used in the text to indicate the measurements of the specimens:

**BL** body length (length from the apex of pronotum to the apex of elytra in dorsal view);

**BW** body width (distance between the humeri, maximal body width);

**HL** head length (length from occiput to the front apex of mandibles);

**HW** head width (distance between the outer margin of eyes in frontal view, maximal head width);

**PL** pronotal length (length from the basal angle to anterior margin, maximal longitudinal length of pronotum);

**PW** pronotal width (distance of the widest portion of the pronotum);

**EL** elytral length (length of the maximal elytral length in dorsal view);

**AL** aedeagus length (length from the apex of aedeagus to the basal margin, maximal aedeagus length);

**AW** aedeagus width (the maximal width of aedeagus);

**SL** spermathecal length (length of the maximal spermathecal length, without duct).

## Taxonomy

### Genus Melixanthus

Taxon classificationAnimaliaColeopteraChrysomelidae

Suffrian, 1854

FDC938FF-9186-5FA1-A015-7F508CF9A92B

Suffrian 1854: 8; Chapuis 1874: l75; Jacoby 1908: 267; Clavareau 1913: 197; Gressitt 1942: 330 353; Chûjô 1954: 187; Gressitt and Kimoto 1961: 169; Tan et al. 1981: 174; Kimoto and Gressitt 1981: 329; Schöller et al. 2010: 606; Medvedev 2012: 162.

#### Type species.

*Melixanthusintermedius* Suffrian, 1854.

#### Synonym.

*Suffrianus* Weise, 1895: 58. Type species: *Cryptocephaluspumilio* Suffrian, 1854.

### Melixanthus menglaensis

Taxon classificationAnimaliaColeopteraChrysomelidae

Duan, Wang & Zhou sp. nov.

C2A184B6-0155-5CB3-9F40-E71664412265

http://zoobank.org/CDAC18DD-5DF4-4115-9CCA-42553FB9E0C2

**Figure 1.**
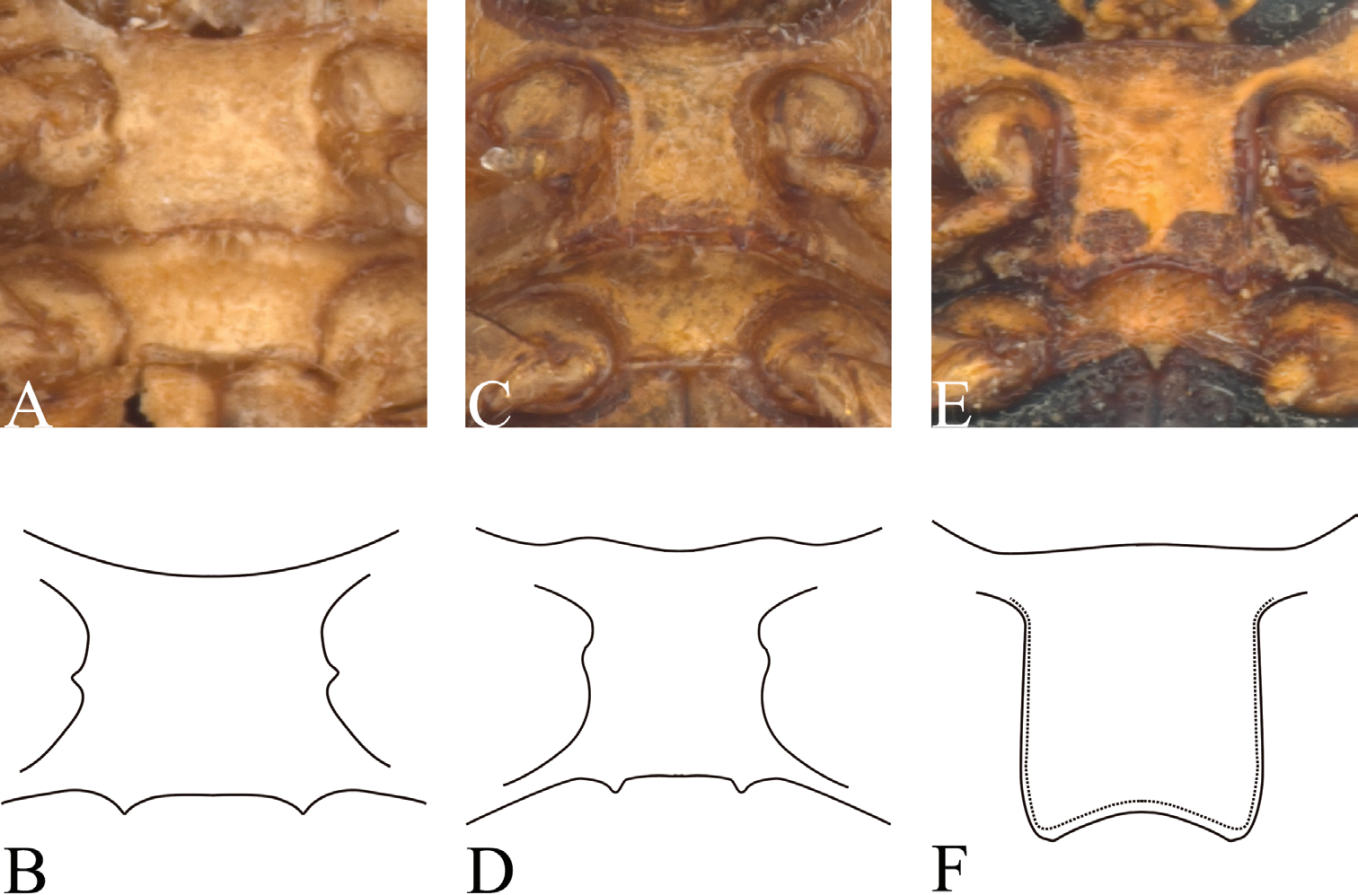
*Melixanthus* prosternum **A, B***M.menglaensis* Duan, Wang & Zhou, sp. nov. **C, D***M.similibimaculicollis* Duan, Wang & Zhou, sp. nov. **E, F***M.bimaculicollis* Baly, 1865.

**Figure 2.**
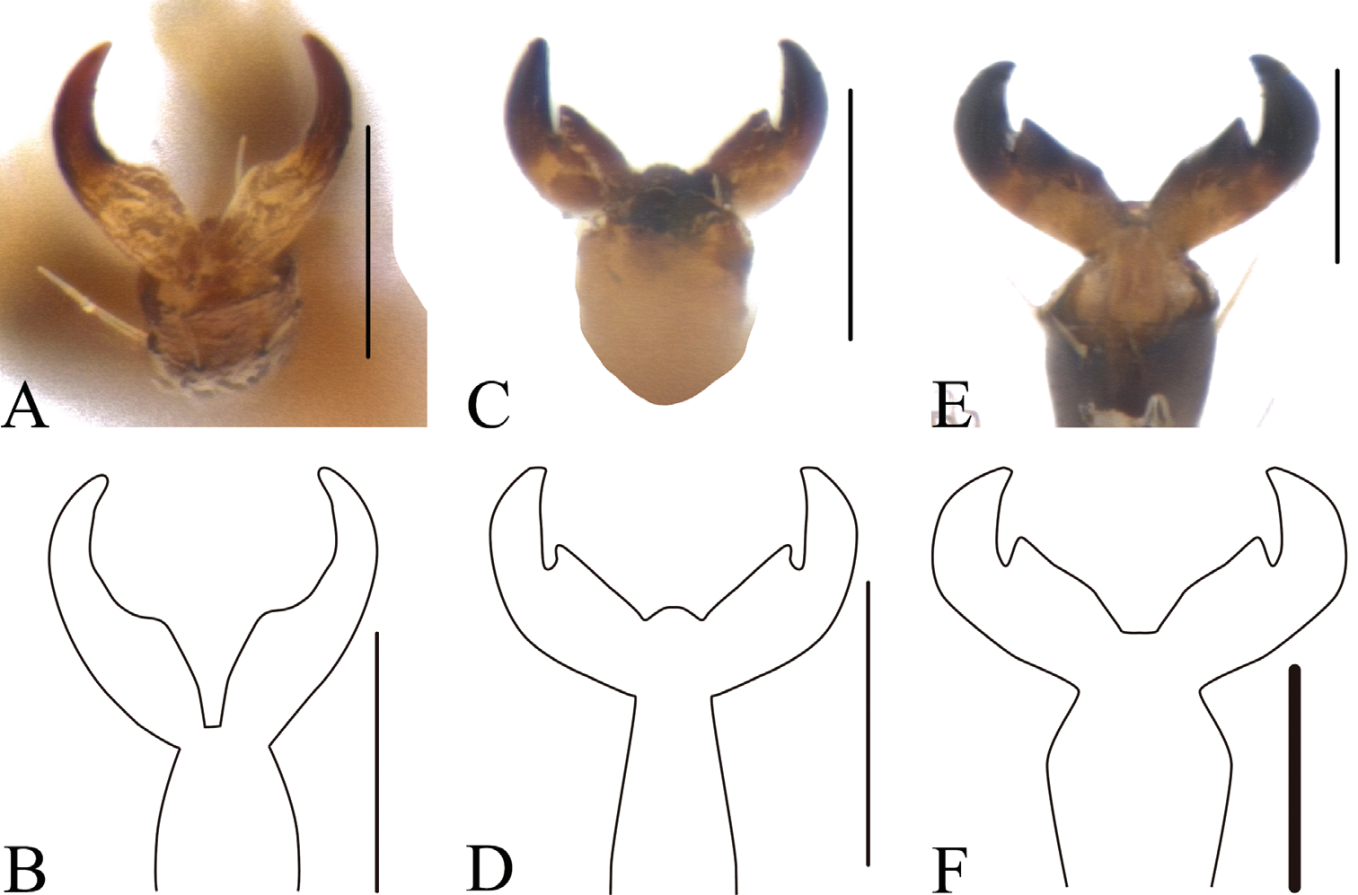
*Melixanthus* claws **A, B***M.menglaensis* Duan, Wang & Zhou, sp. nov. **C, D***M.similibimaculicollis* Duan, Wang & Zhou, sp. nov. **E, F***M.bimaculicollis* Baly, 1865. Scale bars: 0.1 mm.

**Figure 3.**
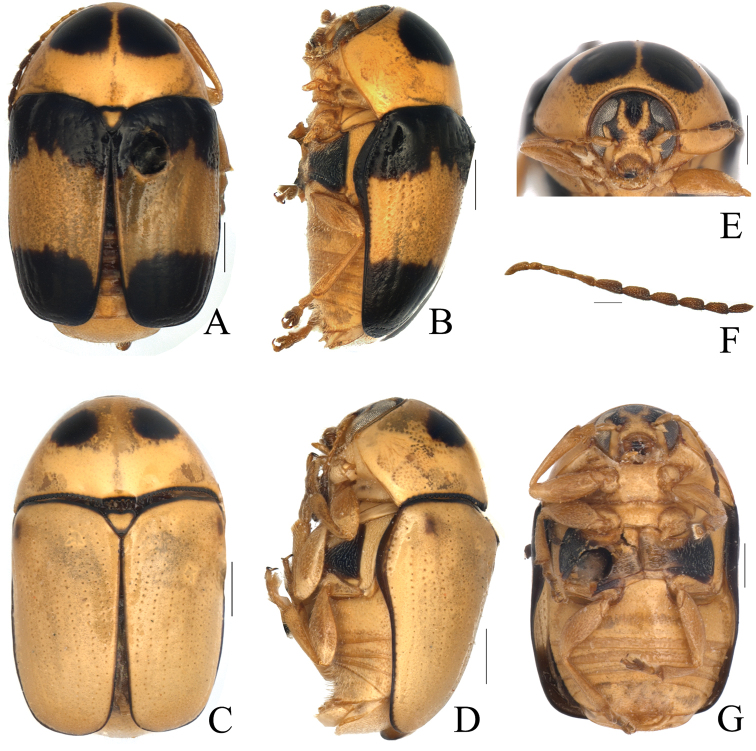
*Melixanthusmenglaensis* Duan, Wang & Zhou, sp. nov. **A, C** habitus **B, D** lateral view of habitus **E** head **F** antennae **G** ventral view of habitus. Scale bars: 0.5 mm (**A–E, G**), 0.2 mm (**F**).

**Figure 4.**
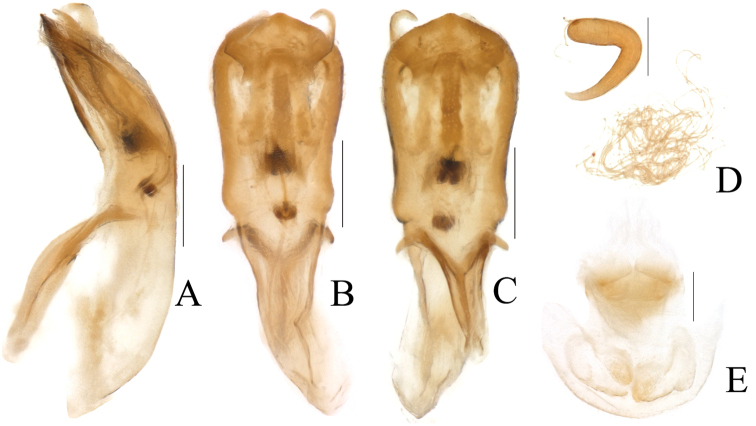
*Melixanthusmenglaensis* Duan, Wang & Zhou, sp. nov. **A** lateral view of aedeagus **B** dorsal view of aedeagus **C** ventral view of aedeagus **D** spermatheca **E** female rectal pad. Scale bars: 0.2 mm.

**Figure 5.**
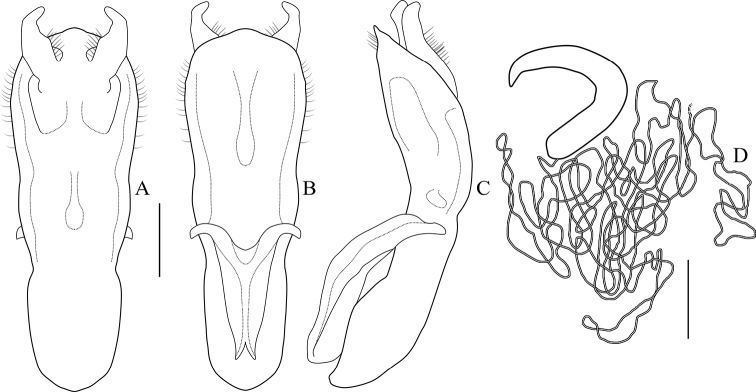
*Melixanthusmenglaensis* Duan, Wang & Zhou, sp. nov. **A** dorsal view of aedeagus **B** ventral view of aedeagus **C** lateral view of aedeagus **D** spermatheca. Scale bars: 0.2 mm.

Figures 1 , 2 , 3 , 4 , 5

#### Type locality.

China: Yunnan Province: Mengla.

#### Type material examined.

***Holotype*:** male, **China: Yunnan Province**: Mengla, Menglun, II–IV.1979, coll. unknown (IZ-CAS). ***Paratypes*: China: Yunnan Province**: 4 males, 2 females, same data as holotype (IZ-CAS); 1 female, Xishuangbanna, Gannanba, 14.III.1957, coll. Shuyong Wang (IZ-CAS); 1 female, Xishuangbanna, Gannanba, 21.III.1957, coll. Shuyong Wang (IZ-CAS); 1 female, Cheli, 9. IV.1955, coll. Fengyu Xue (IZ-CAS). **Vietnam: Tonkin**: 4 males, 3 females, III.1937, coll. unknown (IZ-CAS).

#### Measurements.

BL = 3.25–3.60 mm, BW = 2.02–2.24 mm, HL = 0.91 mm, HW = 0.91 mm, PL = 1.08 mm, PW = 2.02 mm, EL = 2.00 mm, AL = 0.87 mm, AW = 0.27 mm, SL = 0.41 mm.

#### Description.

Body (Fig. 3A–D) elongate, almost cylindrical, rounded anteriorly. Head (Fig. 3E) yellow, vertex with a darkish brown M-shaped marking; antennae (Fig. 3F) with basal 5 segments yellowish brown, terminal 6 segments reddish brown; clypeus yellow; labrum yellowish brown; mandibles darkish brown. Pronotum yellow, with two pitchy brown subtriangular markings along anterior margin. Scutellum yellow and margins black. Elytra pitchy brown, with a yellowish-brown band in middle region, covering about 1/2 of whole region; margins pitchy brown. Sometimes entirely yellow, only margins pitchy brown. Ventral surface yellow, metasternum with a rectangular black marking. Legs and pygidium all yellow.

Head (Fig. 3E) densely and coarsely punctate, flattened on midline, longitudinally impressed on frons and vertex. Eyes kidney-shaped, deeply emarginated; antennal insertions a little more widely separated than superior eye-lobes. Clypeus sparsely punctate, strongly arcuate on anterior margin. Antennae (Fig. 3F) with sparsely long hair, short and slightly broad, reaching humeral tubercle; 1^st^ segment clubbed; 2^nd^ oblong, about half as long as 1^st^; 3^rd^–5^th^ thin, about equal in length, longer than 2^nd^; 6 apical segments moderately thickened, about 2.0–2.2 times as long as wide, last segment pointed apically.

Pronotum (Fig. 3A–D) 1.8 times as wide as long, moderately narrowed and rounded anteriorly; surface strongly convex, impunctate and shining. Scutellum triangular, nearly as long as wide, surface smooth, shining.

Elytron (Fig. 3A–D) parallel-sides, apical margin slightly straight, 2.0 times as long as wide, humeri prominent and glabrous. Disc with regular rows of fine punctures, partly confused near apical slope; interspaces without punctures; epipleurae slightly obliquely placed and seen in lateral view.

Ventral side (Fig. 3G) partly clothed with pubescence. Prosternum (Fig. 1A, B) square, anterior margin slightly concave; basal margin nearly straight, and drawn out into a pair of small denticles. Mesosternum trapeziform, twice as wide as long. Metasternum wrinkled at sutural region and with dense pubescence. Pygidium flat, punctate and pubescent. Claws (Fig. 2A, B) not toothed, thickened basally.

**Aedeagus** (Figs 4A–C, 5A–C) elongate, about 3.2 times as long as wide, clubbed. Apex of median lobe narrower than middle, acute at apex, slightly curved in lateral view; with several pubescence on each side of apex and upper lateral margins, punctate on ventral side of upper middle part. Median orifice with middle sclerite bending inwards above surface. Upper part of median lobe with a pair of sclerotized prominence, exceeding the median lobe. Inner sac rather narrow, arrow-shaped. Tegmen Y-shaped, weakly sclerotized, almost translucent.

**Female.** Body more robust than male; **spermatheca** (Figs 4D, 5D) hook-shaped, bent in a right-angle halfway, slightly acute at apex; duct weakly sclerotized, irregularly coiling 9–12 times. **Rectal sclerites** (Fig. 4E) weakly sclerotized, slightly connected between two rectangular sclerites on ventral side.

#### Distribution.

China (Yunnan); Vietnam (Tonkin).

#### Etymology.

The specific epithet is derived from the name (Pinyin) of the type locality, Mengla.

#### Diagnosis.

This species is similar to *M.bimaculicollis* Baly, 1865, but can be distinguished from that species in having finer punctures on the head, a narrower pronotum, an impunctate scutellum, and fine puncture rows on the elytra, whereas *M.bimaculicollis* has the elytra with distinct punctures and a surrounded by dark ring; its claws are not toothed (Fig. 2A, B) and the basal margin of its prosternum is drawn out into a pair of small, sharp denticles (Fig. 1A, B).

### Melixanthus similibimaculicollis

Taxon classificationAnimaliaColeopteraChrysomelidae

Duan, Wang & Zhou sp. nov.

EEF0DDC7-8F94-5C2A-B8E9-F24D3107F76B

http://zoobank.org/CF48CD44-29B9-4CCA-BB5F-764FA5F15982

**Figure 6.**
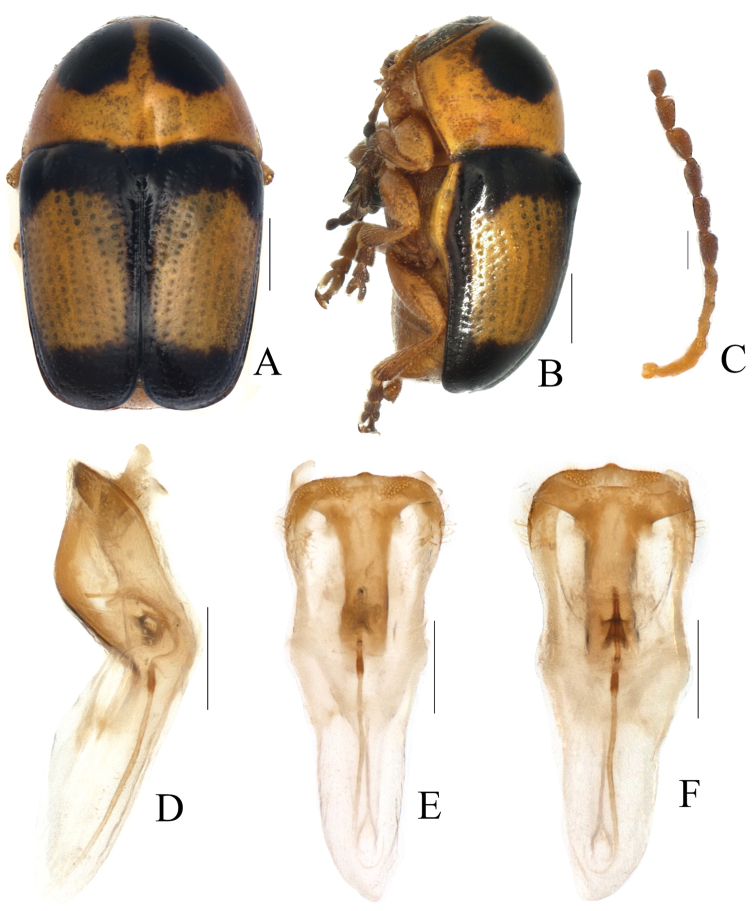
*Melixanthussimilibimaculicollis* Duan, Wang & Zhou, sp. nov. **A** habitus **B** lateral view of habitus **C** antennae **D** lateral view of aedeagus **E** ventral view of aedeagus **F** dorsal view of aedeagus. Scale bars: 0.5 mm (**A, B**), 0.2 mm (**C–F**).

**Figure 7.**
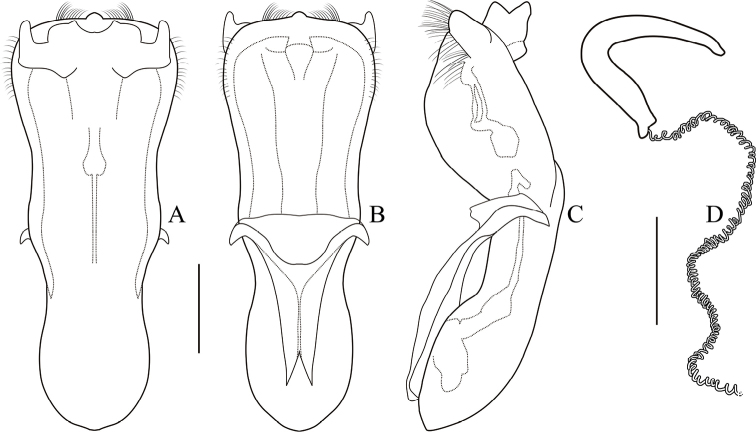
*Melixanthussimilibimaculicollis* Duan, Wang & Zhou, sp. nov. **A** dorsal view of aedeagus **B** ventral view of aedeagus **C** lateral view of aedeagus **D** spermatheca. Scale bars: 0.2 mm.

Figures 1 , 2 , 6 , 7

#### Type locality.

China: Yunnan Province: Cheli.

#### Type material examined.

***Holotype*:** male, **China: Yunnan Province**: Cheli, 9.III.1957, coll. Fuji Pu (IZ-CAS); ***Paratypes*: China: Yunnan Province**: 1 male, 50 km southwest of Mojiang, 30.III.1955, coll. Kryzhanowski (IZ-CAS); 1 female, Longling, 1600 m, 20.V.1955, coll. Kryzhanowski (IZ-CAS).

#### Measurements.

BL = 2.55–2.82 mm, BW = 1.57–1.73 mm, HL = 0.82 mm, HW = 0.85 mm, PL = 0.90 mm, PW = 1.62 mm, EL = 1.81 mm, AL = 0.91 mm, AW = 0.34 mm, SW = 0.39 mm.

#### Description.

Body (Fig. 6A, B) elongate, almost cylindrical, rounded anteriorly. Head yellow, vertex with a darkish brown triangular marking; antennae (Fig. 6C) with basal 5 segments yellowish brown, the rest brown; clypeus yellow; labrum yellowish brown; mandibles reddish brown. Pronotum yellow, and basal margin pitchy brown, forming 2 nearly round pitchy brown markings along anterior margin. Scutellum entirely black. Elytra pitchy black only in basal and apical parts, with a large yellow band in middle region, covering about 2/3 of whole elytron; sutural and lateral margins also pitchy black. Ventral surface yellowish brown.

Head with sparsely pubescence, without punctures, flattened in midline, and with longitudinal shallow groove on frons. Eyes kidney-shaped, deeply emarginated; antennal insertions about equally separated with superior eye-lobes. Clypeus trapeziform, anterior margin concave, without punctures. Antennae (Fig. 6C) long and slightly thin, reaching 1/3 region of elytra; 1^st^ segment clubbed; 2^nd^ oblong, about 1/2 as long as 1^st^; 3^rd^–5^th^ thin, about equal in length, longer than 2^nd^; 6 apical segments moderately thickened, about 1.7–2.0 times as long as wide, last segment pointed apically.

Pronotum (Fig. 6A, B) 1.8 times as wide as long, moderately narrowed and rounded anteriorly; surface strongly convex, impunctate and shining. Scutellum triangular, nearly as long as wide, surface smooth, shining, apically elevated, observable in lateral view.

Elytra (Fig. 6A, B) with humeri prominent and glabrous, widest slightly behind humerus, feebly truncated at apex. Disc with regular rows of coarse punctures; interspace of rows without any punctures; epipleura slightly obliquely placed and observable in lateral view.

Ventral side smooth, partly clothed with pubescence. Prosternum (Fig. 1C, D) square, anterior margin nearly straight; basal margin slightly concave, and drawn out into a pair of small denticles. Mesosternum trapeziform, 1.5 times as wide as long. Metasternum with coarsely sporadic punctures in sutural region and with sparse pubescence. Pygidium flat, punctate and pubescent. Claws (Fig. 2C, D) distinctly toothed, thickened basally.

**Aedeagus** (Figs 6D–F, 7A–C) elongate, about 2.7 times as long as wide, clubbed. Anterior margin of median lobe nearly straight, middle part papillary protruding, strongly curved in lateral view; with several pubescence on each side of apex and upper lateral margins, punctate on apex of median lobe. Median orifice with middle sclerite bending inwards above surface. Upper part of median lobe with a pair of sclerotized prominence, exceeding the median lobe. Inner sac rather narrow, arrow-shaped. Tegmen Y-shaped, weakly sclerotized, almost translucent.

**Female.** Body more robust than male; **spermatheca** (Fig. 7D) hook-shaped, bent at a right-angle halfway, slightly acute at apex. Duct weakly sclerotized, tightly coiled. **Rectal sclerites** absent in specimen studied.

#### Distribution.

China (Yunnan).

#### Etymology.

The specific epithet is derived from the Latin terms *simili*-, *bi*-, *maculi*- and *collis*, to indicate the new species near to *M.bimaculicollis*.

#### Diagnosis.

The new species is similar to *M.bimaculicollis* Baly, 1865, but can be distinguished from it by the smaller body size; head and scutellum without any punctures; slightly narrower pronotum; elytra with finer punctures, and only basal part punctures surrounded by dark ring; basal margin of prosternum (Fig. 1C, D) drawn out into a pair of small sharp denticles. *Melixanthussimilibimaculicollis* is also similar to *M.menglaensis* Duan, Wang & Zhou, sp. nov., but can be distinguished by the following characters: head without punctures; claws toothed (Fig. 2C, D); and body size smaller.

### Melixanthus rufiventris

Taxon classificationAnimaliaColeopteraChrysomelidae

Pic, 1926, new country record from China

2C0B70ED-0790-53FC-BF0E-3BD9196BBAA7

Figures 8 , 9

Pic 1926: 11 (type locality: Tonkin); Kimoto and Gressitt 1981: 333 (Vietnam); Medvedev 2012: 163.

#### Material examined.

**China: Hunan province**: 3 males, 6 females, Shimen country, Hupingshan town, Wangyue lake, 29.93222°N, 110.7776°E, 248 m, 11.X.2014, coll. Jian Yao (IZ-CAS).

#### Measurements.

BL = 2.65–2.93 mm, BW = 1.64–1.80 mm, HL = 0.75 mm, HW = 0.80 mm, PL = 0.92 mm, PW = 1.73 mm, EL = 1.92 mm, AL = 0.64 mm, AW = 0.20 mm.

#### Distribution.

China (Hunan); Vietnam.

**Figure 8.**
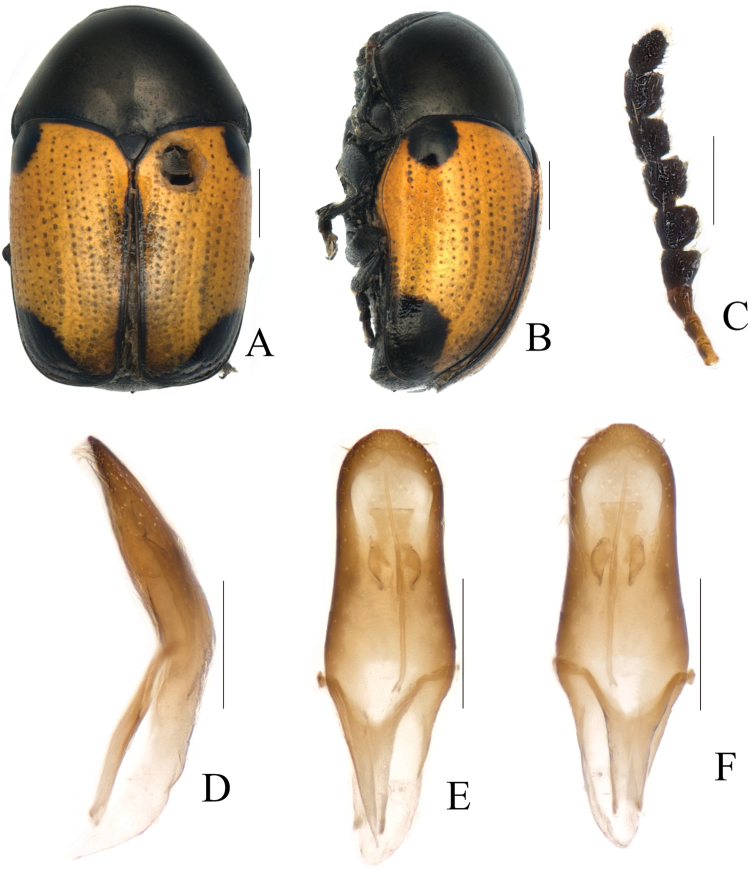
*Melixanthusrufiventris* Pic, 1926 **A** habitus **B** lateral view of habitus **C** antennae **D** lateral view of aedeagus **E** ventral view of aedeagus **F** dorsal view of aedeagus. Scale bars: 0.5 mm (**A, B**), 0.2 mm (**C–E**).

**Figure 9.**
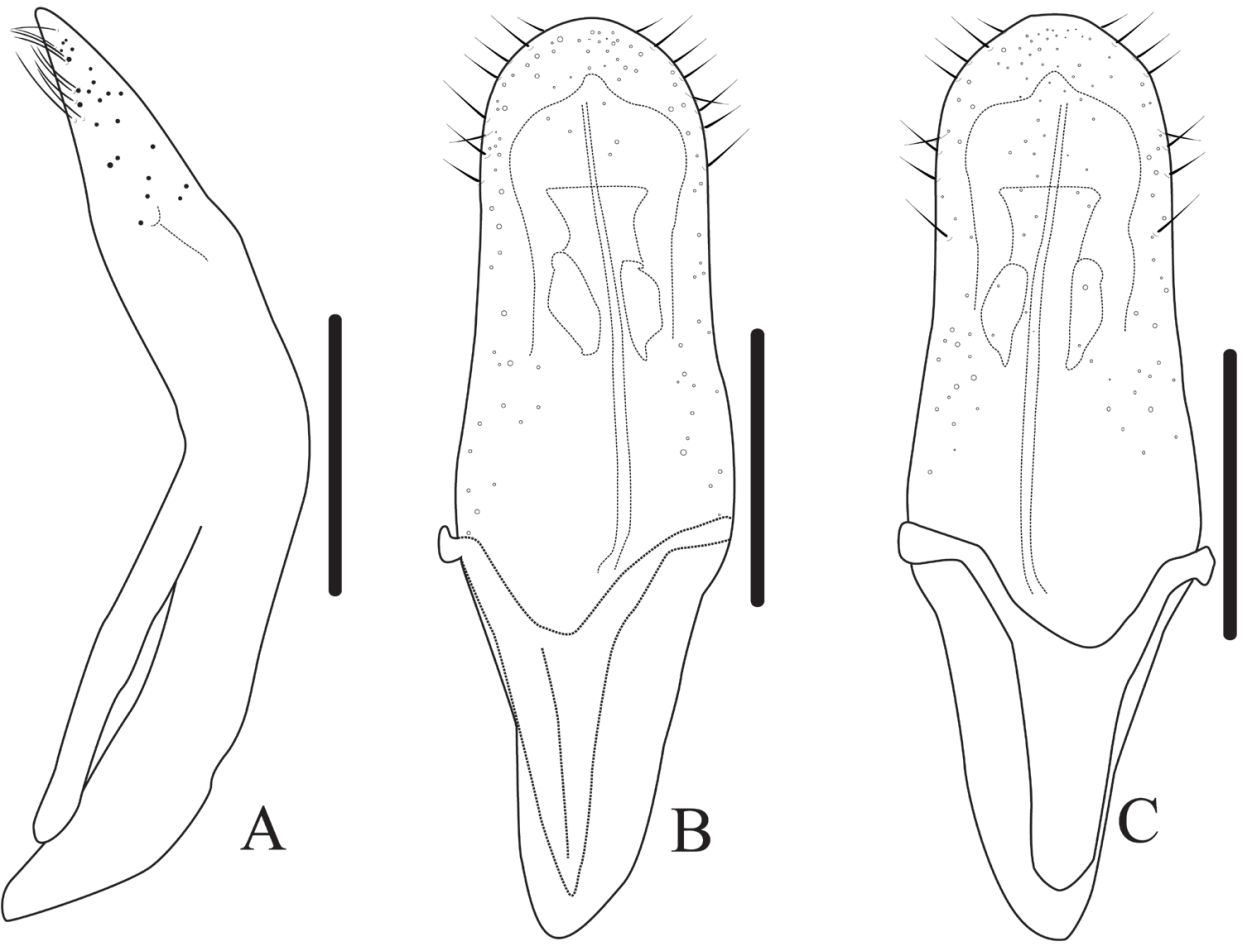
*Melixanthusrufiventris* Pic, 1926 **A** lateral view of aedeagus ventral view of aedeagus **C** dorsal view of aedeagus **D** spermatheca. Scale bars: 0.2 mm.

## Supplementary Material

XML Treatment for Melixanthus

XML Treatment for Melixanthus menglaensis

XML Treatment for Melixanthus similibimaculicollis

XML Treatment for Melixanthus rufiventris
